# Effects of Vitamin D3 Supplementation on Inflammatory Markers in Overweight and Obese Children and Adolescents: A Systematic Review

**DOI:** 10.3390/life15071142

**Published:** 2025-07-20

**Authors:** Maria Krajewska, Ewelina Witkowska-Sędek

**Affiliations:** Department of Paediatrics and Endocrinology, Medical University of Warsaw, 02-091 Warsaw, Poland; ewelina.witkowska-sedek@wum.edu.pl

**Keywords:** vitamin D3 supplementation, children and adolescents, overweight, obesity, low-grade inflammation, C-reactive protein, interleukin-6, tumor necrosis factor-α, leptin, adiponectin, systematic review

## Abstract

Obesity-related low-grade inflammation is a significant factor responsible for the development of metabolic syndrome and chronic diseases, which can begin even in early childhood. Recently, there has been growing interest in the impact of vitamin D3 supplementation on inflammatory markers in overweight and obese individuals; however, findings remain inconsistent. Therefore, we aimed to conduct a systematic review to assess the effects of vitamin D3 supplementation on inflammatory markers in overweight and obese children and adolescents, focused exclusively on the analysis of randomized controlled trials (RCTs) identified by searching PubMed, EMBASE, and Cochrane Library. The results of this study were synthesized and reported following the PRISMA statement. A total of 294 citations were identified through electronic literature searches, of which two RCTs were finally included in our systematic review. We found that vitamin D3 supplementation did not affect the changes in C-reactive protein (CRP), interleukin-6 (IL-6), and tumor necrosis factor-α (TNF-α), but led to a decrease in leptin levels. The small number of studies meeting the inclusion criteria for our systematic review limits the value of the presented results, but also indicates the need for in-depth research on this topic.

## 1. Introduction

Considering the increasing global incidence of overweight and obesity among children, adolescents, and young adults, all research related to the pathophysiology of metabolic disorders and chronic diseases resulting from excess body fat is valuable. Recent data from the World Health Organization shows that in 2022, 8% of children and adolescents aged 5 to 19 were obese, which translates to 160 million young people [1]. Obesity-related low-grade inflammation is a significant factor responsible for the development of metabolic syndrome and chronic diseases, primarily cardiovascular diseases, which can occur even in early childhood [2,3,4]. Excess body fat mass leads to significant changes in adipose tissue metabolism, including increased secretion of pro-inflammatory factors such as interleukins (ILs) (mainly IL-6, IL-8, IL-1β, IL-17), leptin, tumor necrosis factor-α (TNF-α), and decreased levels of adiponectin and anti-inflammatory ILs (e.g., IL-4, IL-10, and IL-13) [5,6,7]. These mediators are involved in the mutual interactions between the immune and metabolic systems, leading to the development of insulin resistance, hyperglycemia, dyslipidemia, and hypertension, resulting in an increased risk of atherosclerotic cardiovascular disease and type 2 diabetes [8,9,10]. In recent years, many researchers have investigated the factors that may reduce the severity of obesity-related chronic low-grade inflammation, thus minimizing and/or delaying the development of complications associated with excess body fat. Vitamin D seems to be one of those factors [11,12]. Vitamin D receptors (VDR) are present in almost all immune system cells as well as in the epithelial cells of the intestines, pancreas, prostate, lungs, and cardiomyocytes. Furthermore, some immune cells, such as dendritic cells, macrophages, and B and T cells, can synthesize calcitriol through the expression of 1α-hydroxylase. Through these mechanisms, vitamin D influences the differentiation, maturation, and metabolism of immune system cells and modulates the production of pro-inflammatory and anti-inflammatory cytokines, resulting in its anti-inflammatory and antioxidant effects [13,14,15,16,17]. Clinical studies confirm that vitamin D status influences chronic inflammation in adipose tissue [18,19,20,21]. There is also some evidence that vitamin D status is inversely correlated with serum C-reactive protein (CRP) levels [19,22]. It is well known that vitamin D deficiency is common in both pediatric and adult populations, especially among overweight and obese individuals [11,23].

Studies analyzing the immunological and metabolic effects of vitamin D supplementation in obese participants differ significantly in terms of study design, age, and number of participants, doses of vitamin D, as well as the types of assessed parameters, including those related to chronic inflammation. The results of studies are often inconsistent and ambiguous.

This systematic review aims to evaluate the effects of vitamin D3 supplementation on inflammatory markers in obese children and adolescents. To ensure the high scientific value of our study, we focused exclusively on the analysis of randomized controlled trials (RCTs).

## 2. Materials and Methods

This review was performed according to the 2020 Preferred Reporting Items for Systematic Reviews and Meta-Analyses (PRISMA) guidelines [24] and preregistered in the International Prospective Register of Systematic Reviews—PROSPERO (CRD420251022458). Available from: https://www.crd.york.ac.uk/PROSPERO/view/CRD420251022458 (accessed on 19 May 2025).

### 2.1. Search Strategy

A systematic search strategy was conducted in three different databases (PubMed, Embase, Cochrane Library) to detect relevant RCTs published from January 2010 until April 2025 with a restriction to the English language. The following search terms were used to select primary articles, containing MeSH and non-MeSH terms:

(“Vitamin D”[Mesh] OR “Vitamin D” OR cholecalciferol OR “25(OH)D3” OR “vit D” OR “vitamin D3” OR “25-hydroxy-vitamin D” OR “25-hydroxy vitamin D” OR “25-hydroxyvitamin D” OR “(25(OH)D)”) AND (administration OR supplementation) AND (“Pediatric obesity”[Mesh] OR “pediatric obesity” OR “childhood obesity” OR “obesity in children” OR “obesity in adolescents” OR “children with obesity” OR “adolescents with obesity” OR “obese children” OR “obese adolescents” OR “obese children” OR (“Obesity”[Mesh] AND (preschool OR child OR kid OR kids OR minors OR boy OR boys OR girl* OR childhood OR boyhood OR pediatric OR paediatric OR preteen OR schoolchild* OR underage OR adolescent OR juvenile OR teenager OR pubescen*)) OR (“Obesity, Morbid”[Mesh] AND (preschool OR child OR kid OR kids OR minors OR boy OR boys OR girl* OR childhood OR boyhood OR pediatric OR paediatric OR preteen OR schoolchild* OR underage OR adolescent OR juvenile OR teenager OR pubescen*)) OR (“Obesity, Abdominal”[Mesh] AND (preschool OR child OR kid OR kids OR minors OR boy OR boys OR girl* OR childhood OR boyhood OR pediatric OR paediatric OR preteen OR schoolchild* OR underage OR adolescent OR juvenile OR teenager OR pubescen*)) OR (Obesity AND (preschool OR child OR kid OR kids OR minors OR boy OR boys OR girl* OR childhood OR boyhood OR pediatric OR paediatric OR preteen OR schoolchild* OR underage OR adolescent OR juvenile OR teenager OR pubescen*)) OR (“Overweight”[Mesh] AND (preschool OR child OR kid OR kids OR minors OR boy OR boys OR girl* OR childhood OR boyhood OR pediatric OR paediatric OR preteen OR schoolchild* OR underage OR adolescent OR juvenile OR teenager OR pubescen*)) OR (Overweight AND (preschool OR child OR kid OR kids OR minors OR boy OR boys OR girl* OR childhood OR boyhood OR pediatric OR paediatric OR preteen OR schoolchild* OR underage OR adolescent OR juvenile OR teenager OR pubescen*)) OR (“Adipose Tissue”[Mesh] AND (preschool OR child OR kid OR kids OR minors OR boy OR boys OR girl* OR childhood OR boyhood OR pediatric OR paediatric OR preteen OR schoolchild* OR underage OR adolescent OR juvenile OR teenager OR pubescen*)) OR (“adipose tissue” AND (preschool OR child OR kid OR kids OR minors OR boy OR boys OR girl* OR childhood OR boyhood OR pediatric OR paediatric OR preteen OR schoolchild* OR underage OR adolescent OR juvenile OR teenager OR pubescen*))).

The description of search strategies in Embase and Cochrane Library databases is provided in Appendix A, Table A1.

To avoid missing relevant studies, the reference lists of all included articles were checked to identify additional relevant papers.

### 2.2. Eligibility Criteria

Studies that fulfilled the following criteria were included:

(1) Population: overweight or obese children and adolescents at the age of 9–19 years, based on the 2000 Center for Disease Control growth charts [25], a body mass index (BMI) ≥ 85th percentile was defined as overweight, and a BMI ≥ 95th percentile as obesity
(2) Intervention: oral supplementation of vitamin D3 (cholecalciferol), regardless of the dose or duration of administration
(3) Comparison: placebo
(4) Outcome: the effects of oral vitamin D3 supplementation on inflammatory markers in the blood
(5) Study: RCT

Exclusion criteria:

(1) Case reports, case series, and observational studies
(2) Vitamin D2 (ergocalciferol) or 1,25(OH)2D3 (calcitriol) supplementation
(3) Studies focused on participants with acute or chronic conditions (e.g., endogenous obesity, Cushing’s disease, asthma)
(4) Number of participants less than 10
(5) Not English articles

Population data from conference proceedings and abstracts were not considered if they were not published as full articles.

### 2.3. Study Selection and Data Extraction

Two independent researchers scanned the titles and abstracts of the obtained articles according to established eligibility criteria to exclude irrelevant studies. The full text of the remaining articles was then thoroughly evaluated to select only relevant studies. Using a pre-designed data collection form, the data were then extracted by two independent reviewers.

If sufficient data on the study were not available, an attempt was made to contact the corresponding authors.

The following information was extracted from the qualified trials: first author’s name, year of publication, country of the study, number of individuals in the intervention arm and the control arm, the age range of participants, study design, 25(OH)D levels at baseline and during/after the intervention, dose, and duration of intervention, the influence on the inflammatory biomarkers including CRP, IL-6, TNF-α, leptin, and adiponectin.

### 2.4. Quality Assessment

Quality assessment of selected studies was carried out using the Cochrane risk of bias tool, which evaluates the article in terms of seven domains: random sequence generation, allocation concealment, blinding of participants and personnel, blinding of outcome assessment, incomplete outcome data, selective reporting, and other sources of bias. The assessment of the risk of bias for each domain is presented as “low risk”, “high risk”, or “unclear risk” based on the instructions of the Cochrane Collaboration [26]. The studies in which all domains were graded as low risk of bias were considered to be of low risk of bias. Those with low or unclear risk of bias for all key domains were of unclear risk of bias. Studies with a high risk of bias for one or more key domains were considered to be of high risk of bias. Two independent reviewers assessed selected studies according to above mentioned guidelines.

## 3. Results

### 3.1. Study Selection

**Figure 1:**
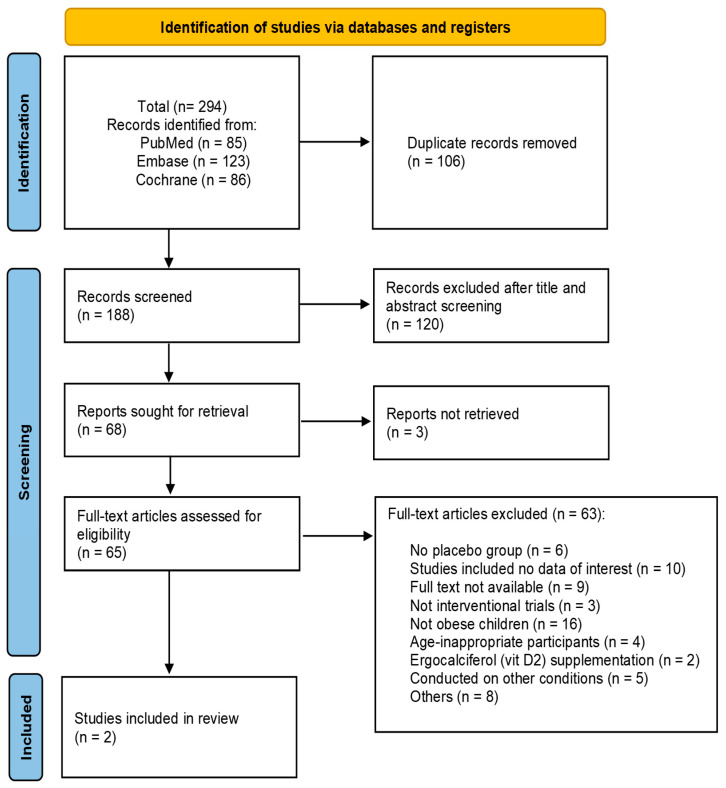
Flow diagram of study selection.

A total of 294 unique citations were identified through electronic literature searches using our initial search strategy, of which 188 remained after duplicates were deleted. After titles and abstracts screening, 120 records were excluded, leaving 68 reports, of which 3 reports were not retrieved. Sixty-five records were eligible for further full-text review. Of these articles, 63 were removed according to inclusion and exclusion criteria. Finally, 2 studies [27,28] were included in our systematic review. The flowchart of study selection is presented in Figure 1.

### 3.2. Characteristics of Included Studies

The included studies were conducted in the United States of America (USA) and published between 2013 and 2014. Participants were in total of 79 obese children and adolescents, both sexes, aged 9–19. Cholecalciferol supplementation lasted from 12 weeks to 6 months, with a 2000 to 4000 IU/day dose. Mean baseline BMI ranged from 33.9 ± 5.3 kg/m^2^ to 39.5 ± 5.1 kg/m^2^. Characteristics of the included studies are demonstrated in Table 1.

### 3.3. Risk of Bias Assessment

**Figure 2:**
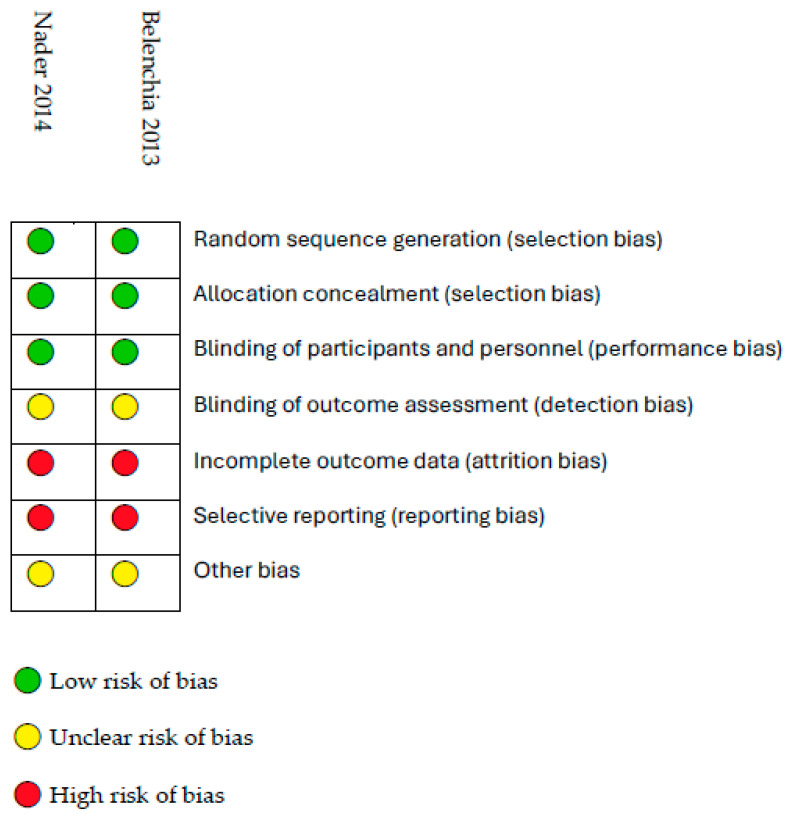
Quality assessment of included studies [27,28].

The risk of bias assessment of the included studies is presented in Figure 2. All the studies provided sufficient information about random sequence generation, allocation concealment, and binding of participants and personnel. These two reports did not provide a satisfactory description of the blinding process of outcome assessment and showed a high risk of bias in both categories: incomplete outcome data and selective reporting.

### 3.4. Effects of Cholecalciferol on C-Reactive Protein Levels

The effect of cholecalciferol on CRP was reported in the trial with 35 obese adolescents (vitamin D group: 18, placebo group: 17). There were no significant changes in CRP between the vitamin D3 and placebo groups, as well as within these groups, from baseline at either 3 or 6 months.

The effect of high sensitivity C-reactive protein (hs-CRP) was presented in the study with 44 obese adolescents (vitamin D3 group: 20, placebo group: 24). No changes in hs-CRP in the vitamin D3 group were found compared with the placebo group after 12 weeks.

### 3.5. Effects of Cholecalciferol on IL-6 and TNF-α

The effect of cholecalciferol on IL-6 and TNF-α was reported in the trial with 35 obese adolescents (vitamin D3 group: 18, placebo group: 17). There were no significant differences between the vitamin D3 and placebo groups or within these groups in IL-6 and TNF-α levels, from baseline at either 3 or 6 months.

### 3.6. Effects of Cholecalciferol on Leptin and Adiponectin Levels

The effect of cholecalciferol on leptin and adiponectin was reported in one trial with 35 obese adolescents (vitamin D3 group: 18, placebo group: 17). After 3 months, there was no change in leptin, adiponectin, and the leptin to adiponectin ratio (L/A ratio). However, after 6 months, a significantly lower level of leptin (*p* = 0.023) and the L/A ratio (*p* = 0.009) in the vitamin D3 group was noticed. After 6 months, the L/A ratio was also significantly lower (*p* = 0.045) in the vitamin D3 group compared with the placebo group.

## 4. Discussion

In the current study, we investigated the effect of vitamin D3 supplementation on inflammatory markers in overweight and obese children and adolescents. We found that from January 2010 to April 2025, only two RCTs have been published in this field in the pediatric population. Both studies evaluated the impact of cholecalciferol supplementation on CRP levels [27,28]. The study conducted by Belenchia et al. [27] additionally examined changes in IL-6, TNF-α, leptin, and adiponectin during vitamin D3 supplementation. None of these studies confirmed significant changes in inflammatory markers in obese adolescents during vitamin D3 supplementation, although a statistically significant increase in serum 25(OH)D levels was observed.

It has been confirmed that, in addition to its role in energy storage, adipose tissue also participates in many metabolic and immunological processes. Chronic inflammation is closely related to excess fat mass and is characterized by an imbalance between pro-inflammatory and anti-inflammatory processes, abnormal levels of adipokines, and excessive production of acute-phase reactants [29,30]. The potential benefits of vitamin D supplementation in obese individuals have been intensively studied in recent years. The analysis of the relationship between vitamin D status and selected markers of chronic inflammation usually constitutes part of broader studies on the impact of vitamin D supplementation on body composition and metabolism in obese individuals, mainly in adults. Most studies focus on changes in markers of cardiometabolic risk, such as total cholesterol, low-density lipoprotein cholesterol, high-density lipoprotein cholesterol, triglycerides, blood pressure, and selected anthropometric parameters such as BMI and waist circumference [31,32]. The results presented in the literature are often inconsistent, and most studies are not RCTs.

Our systematic review revealed that since January 2010, only two RCTs have been published on the effects of cholecalciferol supplementation in obese children and adolescents [27,28]. We also found three other studies that, unfortunately, did not meet the inclusion criteria for our systematic review due to the lack of a placebo group [33] or supplementation with vitamin D2 (ergocalciferol) rather than vitamin D3 (cholecalciferol) [34,35]. Although our analysis identified only two RCTs that met the inclusion criteria, we decided to present our findings, considering the importance of the topic and emphasizing the need for further research in this area. The study conducted by Belenchia et al. [27], published in 2013, examined the effects of 6 months of supplementation with cholecalciferol at a dose of 4000 IU/day on fasting glucose levels, fasting insulin, CRP, IL-6, TNF-α, leptin, and adiponectin. The mean baseline serum 25(OH)D concentration in the vitamin D3 group was 19.2 ± 6.3 ng/mL, and after 6 months of supplementation with cholecalciferol, 93% of participants became sufficiently supplied with vitamin D. The authors reported a significant inverse correlation between the change in 25(OH)D levels over 6 months of cholecalciferol supplementation and the change in the L/A ratio. A significant decrease in the L/A ratio was associated with a significant decrease in serum leptin levels during this period. However, the decrease in leptin levels did not lead to significant changes in serum levels of IL-6 and TNF-α. The level of CRP also did not change significantly in relation to the improvement of vitamin D status. The authors concluded that their findings do not support the hypothesis that increasing vitamin D status reduces inflammation in obese individuals. Furthermore, results presented by Belenchia et al. [27] indicate that vitamin D supplementation reduces the L/A ratio independently of obesity, as there were no changes in BMI *z*-score or waist circumference in the vitamin D3 group. The main role of leptin is to promote satiety and influence energy balance by interacting with the hypothalamus, which modulates the relationships between the central nervous system and metabolic homeostasis. As a result of its role as an anorexigenic and pro-inflammatory factor, leptin is considered a link between the neuroendocrine system and the immune system [36]. Leptin is involved in the upregulation of IL-6 and TNF-α, contributing to obesity-related low-grade inflammation, promoting vascular dysfunction by contributing to hypertension, angiogenesis, and atherosclerosis, and interacting with insulin, affecting glucose and lipid metabolism [37,38,39]. Circulating levels of leptin reflect the amount of energy stored in adipose tissue; thus, obese individuals produce more leptin than non-obese individuals. On the other hand, chronic inflammation can impair leptin action and lead to hypothalamic leptin resistance, disrupting body weight control and leading to an increase in fat tissue mass [40,41,42]. Adiponectin is a well-known anti-inflammatory, anti-atherosclerotic, and insulin-sensitizing factor involved in the regulation of glucose and lipid metabolism, affecting insulin sensitivity, and protecting against type 2 diabetes and cardiovascular diseases [37,39,43,44]. The L/A ratio is considered a sensitive biomarker of early metabolic dysregulation in obese individuals, with greater diagnostic utility compared to measuring leptin or adiponectin separately [45]. Subsequent studies confirmed that the leptin-adiponectin axis plays a pathophysiological role in increased systemic inflammation and oxidative stress observed in patients with metabolic syndrome. The results showed that a reduction in the L/A ratio is associated with a decreased risk of atherosclerosis and a reduction in the risk of certain types of cancer in obese individuals [46,47]. A study published by Nader et al. [28] in 2014 examined the effect of 12 weeks of supplementation with 2000 IU/day of cholecalciferol on serum hs-CRP levels, lipid profile, fasting glucose, and insulin resistance markers. The baseline median of 25(OH)D level in the vitamin D3 group was 25.0 ng/mL. The dosing of cholecalciferol in this study resulted in only a modest increment in serum levels of 25(OH)D compared to the placebo group; the median change during vitamin D3 supplementation was 5.0 ng/mL. The authors reported no beneficial effects of vitamin D3 supplementation and postulated the need for long-term supplementation with higher doses of cholecalciferol [28]. It is well known that excess body fat mass is a significant risk factor for vitamin D deficiency. The main mechanisms involved in obesity-related vitamin D deficiency include decreased bioavailability due to its accumulation in adipose tissue, reduced intestinal absorption, impaired metabolism, decreased liver 25(OH)D synthesis, and the influence of leptin and IL-6 on hepatic VDRs. In addition, a sedentary lifestyle, insufficient sun exposure associated with low outdoor physical activity, and inappropriate vitamin D dietary intake increase the risk of hypovitaminosis D in obese individuals [11,20,48,49,50,51,52,53]. The recommended supplementation doses of vitamin D for obese individuals are usually twice as high as for those with normal body weight [54,55]. According to Global Consensus Recommendations on Prevention and Management of Nutritional Rickets [56], the minimum treatment doses of vitamin D3 for children aged 1 to 12 years are 3000–6000 IU/day, and for children over 12 years old, 6000 IU/day. In our country, the Guidelines for Preventing and Treating Vitamin D Deficiency: A 2023 Update in Poland [54] recommend therapeutic doses of cholecalciferol at 4000 IU/day for children over 1 year of age. Considering the above recommendations, it is worth planning a future RCT study with higher doses of cholecalciferol, involving a larger group of patients with better participant compliance.

We assessed both RCTs that we identified in our systematic review as being at high risk of bias in the area of attrition bias and selective reporting. In our opinion, the main limitations of these studies are the small sample size and poor compliance. In both RCTs, more than 10% of participants in both the vitamin D3 group and the placebo group did not complete the study. In the study conducted by Belenchia et al. [27], the baseline group included a total of 44 obese adolescents, of whom only 35 were included in the primary analysis (completed the baseline visit and at least one of two follow-up visits). Only 66% of participants in the vitamin D3 group and 65% in the placebo group completed the study. The study evaluated outcomes at baseline and after 3 and 6 months of intervention, but the authors provided detailed data only from the first and the last visit. The study by Nader et al. [28] included 58 participants, of whom only 44 completed the study—71% of the vitamin D3 group and 80% of the placebo group. The authors presented baseline results and changes in the assessed parameters after 12 weeks of intervention without detailed data regarding the outcomes.

The small number of studies meeting the inclusion criteria for our systematic review limits the value of the results presented, but also indicates the need for in-depth research on this topic based on large multicenter cohorts of obese children and adolescents. Furthermore, taking into account vitamin D metabolism in obese individuals, it is worth considering the use of higher cholecalciferol doses and a longer duration of supplementation when planning further studies.

## 5. Conclusions

This systematic review provides the most current evidence from RCTs regarding the impact of vitamin D3 supplementation on inflammatory markers in overweight and obese children and adolescents. Supplementation of 2000–4000 IU/day of cholecalciferol for 3 to 6 months does not lead to a satisfactory increase in serum 25(OH)D levels, which may result in no effect on inflammatory markers such as CRP, IL-6, and TNF-α, but may influence adipokine balance, particularly by reducing leptin levels and the leptin to adiponectin ratio.

## Figures and Tables

**Table 1:** Characteristics of the included studies.

References (Authors, Year of Publication, Country)	Subjects/Age/Study Design	Baseline 25(OH)D in the Vitamin D3 Group (ng/mL)	Supplementation Strategy (Dose and Duration)	25(OH)D During/at the End of the Intervention	Evaluation of Inflammatory Biomarkers
Belenchia et al. 2013, USA [27]	35 obese adolescents (n = 18 vitamin D3 group, n = 17 placebo group)/9–19 years/ randomized double-blind, placebo-controlled trial	19.2 ± 6.3	4000 IU/day Duration: 6 months	After 3 months, no subjects in the vitamin D3 group were 25(OH)D deficient. At 6 months, 93% of participants in the vitamin D3 group were 25(OH)D sufficient.	CRP, IL-6, TNF-α, and adiponectin remained unchanged after 3 and 6 months (placebo compared with control group). After 3 months no change in leptin. After 6 months, significant decrease in the leptin to adiponectin ratio in the vitamin D3 group compared with the placebo group.
Nader et al., 2014, USA [28]	44 obese adolescents (n = 20 vitamin D3 group, n = 24 placebo group)/12–18 years/ randomized double-blind, placebo-controlled trial	25.8 ± 5.9	2000 IU/day Duration: 12 weeks	After 12 weeks, 25(OH)D increased in the vitamin D3 group to a median of 31 ng/mL. Ten of the 20 participants in the vitamin D3 group achieved 25(OH)D > 30 ng/mL at 3 months.	No changes in hs-CRP in the vitamin D3 group compared with the placebo group.

## Data Availability

The original contributions presented in this study are included in the article. Further inquiries can be directed to the corresponding author.

## Abbreviations

The following abbreviations are used in this manuscript:

RCT	randomized controlled trial
CRP	C-reactive protein
hs-CRP	high sensitivity C-reactive protein
ILs	interleukins
IL-6	interleukin-6
TNF-α	tumor necrosis factor-alpha
BMI	body mass index
L/A ratio	leptin to adiponectin ratio
VDRs	Vitamin D receptors

## Appendix A

**Table A1:** Search strategy to find articles suitable for inclusion in the systematic review.

PubMed
(“Vitamin D”[Mesh] OR “Vitamin D” OR cholecalciferol OR “25(OH)D3” OR “vit D” OR “vitamin D3” OR “25-hydroxy-vitamin D” OR “25-hydroxy vitamin D” OR “25-hydroxyvitamin D” OR “(25(OH)D)”)
AND
(administration OR supplementation)
AND
(“Pediatric obesity”[Mesh] OR “pediatric obesity” OR “childhood obesity” OR “obesity in children” OR “obesity in adolescents” OR “children with obesity” OR “adolescents with obesity” OR “obese children” OR “obese adolescents” OR “obese children” OR (“Obesity”[Mesh] AND (preschool OR child OR kid OR kids OR minors OR boy OR boys OR girl* OR childhood OR boyhood OR pediatric OR paediatric OR preteen OR schoolchild* OR underage OR adolescent OR juvenile OR teenager OR pubescen*)) OR (“Obesity, Morbid”[Mesh] AND (preschool OR child OR kid OR kids OR minors OR boy OR boys OR girl* OR childhood OR boyhood OR pediatric OR paediatric OR preteen OR schoolchild* OR underage OR adolescent OR juvenile OR teenager OR pubescen*)) OR (“Obesity, Abdominal”[Mesh] AND (preschool OR child OR kid OR kids OR minors OR boy OR boys OR girl* OR childhood OR boyhood OR pediatric OR paediatric OR preteen OR schoolchild* OR underage OR adolescent OR juvenile OR teenager OR pubescen*)) OR (Obesity AND (preschool OR child OR kid OR kids OR minors OR boy OR boys OR girl* OR childhood OR boyhood OR pediatric OR paediatric OR preteen OR schoolchild* OR underage OR adolescent OR juvenile OR teenager OR pubescen*)) OR (“Overweight”[Mesh] AND (preschool OR child OR kid OR kids OR minors OR boy OR boys OR girl* OR childhood OR boyhood OR pediatric OR paediatric OR preteen OR schoolchild* OR underage OR adolescent OR juvenile OR teenager OR pubescen*)) OR (Overweight AND (preschool OR child OR kid OR kids OR minors OR boy OR boys OR girl* OR childhood OR boyhood OR pediatric OR paediatric OR preteen OR schoolchild* OR underage OR adolescent OR juvenile OR teenager OR pubescen*)) OR (“Adipose Tissue”[Mesh] AND (preschool OR child OR kid OR kids OR minors OR boy OR boys OR girl* OR childhood OR boyhood OR pediatric OR paediatric OR preteen OR schoolchild* OR underage OR adolescent OR juvenile OR teenager OR pubescen*)) OR (“adipose tissue” AND (preschool OR child OR kid OR kids OR minors OR boy OR boys OR girl* OR childhood OR boyhood OR pediatric OR paediatric OR preteen OR schoolchild* OR underage OR adolescent OR juvenile OR teenager OR pubescen*)))
Embase
((‘child’/exp OR ‘child’) OR (‘minor (person)’/exp OR ‘minor (person)’) OR adolescen* OR boy OR boys OR boyhood OR child* OR girl OR juvenile OR kid OR kids OR minors OR paediatric OR pediatric OR preschool* OR preteen OR pubescen* OR choolchild* OR teenage? OR underage)
AND
(‘obesity’/exp OR ‘obesity’ OR obes* OR ‘overweight’/exp OR overweight* OR ‘adiposity’/exp OR adiposity OR ‘adipose tissue’/exp OR ‘adipose tissue’)
AND
(‘vitamin d’/exp OR ‘vitamin d’ OR ((‘vitamin’/exp OR vitamin) AND d$) OR ‘colecalciferol’/exp OR ‘colecalciferol’ OR (25 AND hydroxyvitamin AND d$) OR (25 AND hydroxy AND vitamin AND d$) OR cholecalciferol OR cholecalciferol? OR (25 AND oh AND d$) OR (vit AND d$))
AND
(‘supplementation’/exp OR ‘supplementation’ OR ‘administration of drugs, food and chemicals’/exp OR ‘administration of drugs, food and chemicals’)
Cochrane Library
ID Search
#1 MeSH descriptor: [Overweight] explode all trees
#2 MeSH descriptor: [Adolescent] explode all trees
#3 MeSH descriptor: [Pediatrics] explode all trees
#4 MeSH descriptor: [Child] explode all trees
#5 MeSH descriptor: [Cholecalciferol] explode all trees
#6 MeSH descriptor: [Vitamin D] explode all trees
#7 (child* or adolescen* or juvenil* or youth* or preschool* or teen* or preteen* or boy or boys or boyhood or girl or schoolchild* or underage or pubescen* or p?adiatric or minor* or kid or kids):ti,ab,kw
#8 (obesity or obes* or overweight or adiposity or “adipose tissue”):ti,ab,kw
#9 (“vitamin d” or “vitamin D3” or “vit d” or “vit d3” or cholecalciferol or “25 hydroxyvitamin d” or colecalciferol or “25 hydroxy vitamin d” or “25 oh d” or “25 oh d3”):ti,ab,kw
#10 #1 or #8
#11 #2 or #4 or #7
#12 #5 or #6 or #9
#13 supplementation or administration
#14 #10 and #11 and #12 and #13
